# Analysis of LPI-causing mutations on y+LAT1 function and localization

**DOI:** 10.1186/s13023-019-1028-2

**Published:** 2019-03-04

**Authors:** Bianca Maria Rotoli, Amelia Barilli, Filippo Ingoglia, Rossana Visigalli, Massimiliano G. Bianchi, Francesca Ferrari, Diego Martinelli, Carlo Dionisi-Vici, Valeria Dall’Asta

**Affiliations:** 1 Unit of General Pathology, Deptartment of Medicine and Surgery (DiMeC), University of Parma, Via Volturno 39, 43125 Parma, Italy; 2 Division of Metabolic Diseases, Department of Pediatric Specialties, Bambino Gesù Children’s Hospital, Rome, Italy

**Keywords:** eGFP fusion proteins, Arginine transport, Subcellular protein localization, y^+^L transport system, Site-directed mutagenesis

## Abstract

**Background:**

y+LAT1, encoded by SCL7A7, is the protein mutated in Lysinuric Protein Intolerance (LPI), a rare metabolic disease caused by a defective cationic amino acid (CAA, arginine, lysine, ornithine) transport at the basolateral membrane of intestinal and renal tubular cells. The disease is characterized by protein-rich food intolerance with secondary urea cycle disorder, but symptoms are heterogeneous with lung and immunological complications that are not explainable by the CAA transport defect. With the exception of the Finnish founder mutation (c.895-2A > T, LPI_Fin_), LPI-causative mutations are heterogeneous and genotype*-*phenotype correlations have not been found. Here we addressed system y^+^L-mediated arginine uptake in monocytes from three LPI Italian patients and in lymphoblasts carrying the same mutations; in parallel, the genetic defects carried by the patients were reproduced as eGFP-tagged y+LAT1 mutants in transfected CHO cells to define the function and localization protein.

**Results:**

System y^+^L activity is impaired in monocytes isolated from all LPI patients, and in CHO cells transfected with the three eGFP-y+LAT1 mutants, but not in lymphoblasts bearing the same mutations. The analysis of protein localization with confocal microscopy revealed that the eGFP-tagged mutants were retained inside the cytosol, with a pattern of expression quite heterogeneous among the mutants.

**Conclusions:**

The three mutations studied of y+LAT1 transporter result in a defective arginine transport both in ex vivo (monocytes) and in vitro (CHO transfected cells) models, likely caused by the retention of the mutated proteins in the cytosol. The different effect of y+LAT1 mutation on arginine transport in monocytes and lymphoblasts is supposed to be due to the different expression of SLC7A7 mRNA in the two models, supporting the hypothesis that the impact of LPI defect largely depends on the relative abundance of LPI target gene in each cell type.

**Electronic supplementary material:**

The online version of this article (10.1186/s13023-019-1028-2) contains supplementary material, which is available to authorized users.

## Background

Lysinuric protein intolerance (LPI; MIM 222700) is a recessively inherited autosomal disease caused by a defective cationic amino acid (CAA: arginine, lysine, ornithine) transport at the basolateral membrane of intestinal and renal tubular cells [1].

From a clinical point of view, the disease manifests with protein-rich food intolerance and secondary urea cycle disorders; symptoms are heterogeneous, including failure to thrive, recurrent vomiting, hepatosplenomegaly, osteoporosis, lung involvement, kidney failure, hematologic abnormalities and immunological disorders. The major incidence of the disease (1/60,000 births) has been observed in Finland, but also in the South of Italy and the North of Japan the prevalence is high; European patients typically originate from Mediterranean countries, especially from North Africa and Turkey [2].

The gene causing LPI, identified at the end of the nineties in SLC7A7 (solute carrier family 7A member 7; MIM #603593), encodes for y+LAT1 protein [3, 4]. This latter is one of the alternative subunits composing the heterodimeric amino acid transporters belonging to transport system y ^+^ L; these transporters are accounted for by a light subunit (y + LAT1 or y + LAT2 coded by SLC7A6) and a glycoprotein (4F2hc/CD98hc) that is necessary for the correct expression of the transporter at membrane level [5]. System y^+^L selectively transports CAA in the absence of sodium, while it requires the cation to interact with neutral amino acids (leucine, glutamine); operatively, it works as an antiport coupling the efflux of CAA to the influx of neutral amino acids and sodium [6]. System y^+^L was first described in erythrocytes, then in placenta, platelets, skin fibroblasts, hepatocytes, small intestine, and kidney; in polarized epithelia it is mainly located onto the basolateral cell membrane, where it mediates the transport of cationic amino acids from the renal and intestinal epithelia toward the bloodstream [7]. This feature of the transporter gains particular relevance when addressing the physiopathology of LPI and, particularly, of its classical hallmarks: normal-to-low plasma levels and leakage of cationic amino acids in the urine are, indeed, directly referable to both a defective intestinal absorption and an increased loss of the amino acids in the kidney consequent to SLC7A7/y+LAT1 mutation; similarly, clinical signs observed upon protein ingestion, such as nausea, vomiting, and, ultimately, episodes of hyperammonemia can be easily explained by an impairment of urea cycle due to the deficiency of intermediates, mainly arginine, in the blood. Conversely, little is known about the molecular and cellular mechanisms responsible for the life-threatening extra-renal complications of the disease, such as those affecting lung and immune system, although we recently provided evidences sustaining a central role for the mononuclear phagocyte system [8–10].

LPI-causative mutations belong to variable mutation types and are spread along the entire gene, indicating the absence of any mutational hot spot in the SLC7A7 region. Thus far, more than 50 different LPI-causing mutations have been described in more than 142 patients from 110 independent families [1, 11]; the mutational heterogeneity reaches the highest level in Southern Italy, where 12 different mutant alleles were identified in 18 independent families, while Finnish and Japanese patients typically display the founder variants c.895-2A > T (LPI_Fin_) [12] and c.1228C > T [13], respectively.

All but one of the known LPI-causing mutations have been shown to completely abolish system y^+^L activity, while the remaining significantly reduces it [13]. In past years studies have been conducted on eight of these mutations to verify a correlation linking the genetic defect and the subcellular location of mutated y+LAT1 protein, so as to explain the impairment of transport activity; results obtained in in vitro models of transfected *X. laevis* oocytes [14], HEK293 cells [15], and MDCK cells [16], suggested that y+LAT1 frameshift mutants do not reach plasma membranes, while missense mutations more likely cause a functional impairment of the protein despite its proper location.

Here, we addressed the effects of different LPI-causing mutations on system y^+^L activity in monocytes isolated from three Italian patients; in parallel, we reproduced the genetic defects in vitro by expressing the mutant y + LAT1 in transfected CHO cells (Chinese Hamster Ovary cells) so as to evaluate the function and subcellular localization of the protein.

## Material and methods

### Cell models

#### Monocytes from LPI patients

Human monocytes were isolated from blood samples of three LPI subjects (LPI_1_, LPI_2_, and LPI_3_) and three age-matched healthy donors (cont_1_, cont_2_ and cont_3_), according to the standard procedure [9]; blood samples were taken at the same time for control subjects and LPI patients. Briefly, 5–10 ml of heparinized blood were diluted with PBS, layered on Lympholyte gradient medium (Cedarlane Laboratories, Celbio, Italy) and centrifuged at 800 g for 20 min at 20 °C; the isolated peripheral blood mononuclear cells (PBMC) were collected and seeded in RPMI1640 growth medium added with 10% endotoxin-free Fetal Bovine Serum (FBS), 2 mM glutamine and antibiotics (penicillin 100Ul/ml, streptomycin 100 μg/ml). After 20-min incubation at 37 °C in an atmosphere at 5% CO2, non-adherent cells were removed with vigorous washes, while adherent monocytes were used immediately. Viability of monocytes was assessed through Trypan blue exclusion and was > 98% in all cases; CD14 expression was greater than 90% (not shown). LPI patients were followed and enrolled at the “Bambino Gesù” Children’s Hospital in Rome (Italy). The study was approved by the local Ethic Committee (approval #13922, 13/04/2017); written informed consent was obtained from all patients and control volunteers.

The first patient (LPI_1_) is an Italian male, currently 5 years old. The patient was found to be homozygous for the c.726G > A transition in exon 5; this nonsense mutation leads to the synthesis of a mutated protein, lacking 6 out of 12 transmembrane domains (p.W242X) [13]. The boy presented since birth with failure to thrive. Routine blood tests performed at 2 months showed a significant increase in LDH (1657 IU/L; normal values, nv: 125–243), slight increase in AST (75 IU/L; nv: 10–34) and gamma-GT (202 IU/L; nv: 12–64). Additional blood tests confirmed increase of LDH (2308–4700 IU/L; nv: 120–300), AST (106–110 IU/L; nv: 16–55), and showed mild hyperammonemia (122 mmol/l; nv: < 30), hyperferritinemia (up to 5320 ng/ml, nv: 22–275). Metabolic tests showed persistent increase of citrulline (83–91 μmol/L; nv: 11–51) and glutamine (1120 μmol/L, nv, 200–800) and decreased levels of cationic amino acids (arginine 20 μmol/L, nv: 30–90; lysine 41–56 μmol/L, nv: 80–300, ornithine 12–18 μmol/L, nv: 50–200). He was put after diagnosis on a hypoproteic diet (1.5 g/kg/day) and pharmacological treatment with citrulline (100 mg/kg/day) and phenylbutyrate (100 mg/kg/day). He never experienced episodes of hyperammonemia or metabolic decompensation. He now manifest poor growth, but a normal neurocognitive development; several abdomen ultrasound scans performed during the follow up documented an enlarged, hyperechogenic liver and splenomegaly. Chest X-ray displays signs of interstitial pneumopathy; urine analysis was unremarkable.

Patient LPI_2_ is a 44 years old Italian female, carrying the homozygous mutation c.1185_1188delTTCT in exon 9 (p.S396LfsX122); the resulting frameshift abolishes the termination codon at position 1775 and introduces a new one at position 1790 [17]. Clinically, she presents with a very mild phenotype with normal physical and intellectual development and aversion for protein-rich foods. Before diagnosis, she experienced recurrent episodes of hyperammonemia with abnormal behavior and lethargy. She remained in good clinical condition under hypoproteic diet (0.5 g/kg/day) and treatment with citrulline (100 mg/kg/day) and sodium benzoate (50 mg/kg/day). The patient was then hospitalized at 42 years of age for an episode of hyperammoniemic encephalopathy, due to bad compliance to the therapy and the diet. At admission, blood ammonia level was 241 mmol/l; a brain CT scan documented bilateral para trigonal hypodensity and globus pallidum calcifications. Sodium benzoate and citrulline therapy were restarted as emergency treatment, with improvement of the state of consciousness and progressive reduction of ammonia after 48 h. During the follow up, chest X-rays displayed signs of interstitial pneumopathy; a pulmonary TC scan documented multiple parenchymal nodularities. Mild proteinuria was documented at urinary function tests. At the last metabolic control she showed normal ammonia levels, high citrulline (75 μmol/L), low arginine (14 μmol/L) and ornithine (24 μmol/L) with normal lysine (91 μmol/L), and increase of glutamine (2100 μmol/L); phenylbutyrate (100 mg/k/die) was therefore added.

Patient LPI_3_ is a 6 years old Italian female, first daughter of consanguineous parents of Indian/Pakistan ancestry, actually aged 7; she carries a homozygous deletion in exons 1–3 (c.1–499del), detected by the Medical Genetics Department at the “Bambino Gesù” Children’s Hospital in Rome (Italy) through the molecular analysis of the coding sequence and the intron-exon junctions of SLC7A7 gene in DNA isolated from peripheral blood sample. She showed since 2 years of life failure to thrive and protein aversion, along with psychomotor delay, easy fatigability and frequent complain of muscle pains in the lower limbs. At 4 years of age, she was admitted for an episode of persistent fever and respiratory infection. A chest X-ray was performed, which showed mild interstitial lung disease. Blood chemistries during hospitalization showed hypoalbuminemia, and increased LDH and ferritin levels (4146 ng/ml). Blood gas studies and urinary function tests documented renal tubular acidosis with glucosuria and phosphaturia and increased β2 microglobulin. The analysis of plasmatic amino acids revealed low concentrations of lysine (59 μmol/L), arginine (19 μmol/L), and ornithine (19 μmol/L), and high glutamine (1189 μmol/L), citrulline (116 μmol/L), alanine (428 μmol/L), and glycine (386 μmol/L) levels. Hepatomegaly and polysplenia were found at abdomen ultrasound scans. She was then diagnosed with LPI and put on a hypoproteic diet (1.5 g/kg/day), added with citrulline (100 mg/kg/day) and sodium benzoate (150 mg/kg/day) supplemented with sodium bicarbonates, vitamins, carnitine and phosphate solution. Under this therapy, marked improvement in clinical picture, growth parameters, biochemical abnormalities, and partial reduction of hepatomegaly was observed.

#### Lymphoblasts from LPI patients

Lymphoblasts had been kindly provided by Dr. G. Sebastio (Department of Paediatrics of the University Federico II of Naples; Italy); they were obtained from lymphocytes isolated from healthy donors and LPI patients different from those mentioned above (see section *Monocytes from LPI patients*), although carrying the same SLC7A7 mutations described for LPI_1_ and LPI_3_ (see above). Cells were routinely grown in RPMI1640 medium added with 10% FBS, 1 mM sodium pyruvate, 2 mM glutamine and antibiotics (penicillin 100Ul /ml, streptomycin 100 μg/ml) and maintained under physiological conditions (37.5 °C, 5% CO_2_, 95% humidity) in exponential growth phase, at a density ranging between 5 × 10^5^ and 2.5 × 10^6^ cells/ml.

#### CHO cells

Chinese Hamster Ovary (CHO) cells were employed to reproduce in vitro the LPI defects LPI_1,_ LPI_2_, and LPI_3_. Missense mutation c.1001 T > G in exon 8 was also reproduced (here named LPI_4_), since the resulting protein (p.L334R) is known to correctly localize onto the membrane [14]. Cells were routinely grown in Ham’s F12 growth medium supplemented with 6% fetal bovine serum (FBS), 2 mM glutamine and 1% penicillin/streptomycin, and maintained at 37 °C in an atmosphere at 5% CO2.

For cell transfection, expression vectors for both SLC3A2/4F2hc and SLC7A7/y + LAT1 were required, so as to guarantee the correct expression, localization and function of y + LAT1 transporter in transfected cells [5]. Plasmids for wild type (w/t) sequences were generated through insertion of the corresponding Open Reading Frame (ORF) sequences in expression cloning vectors by GeneCopoeia™ (Tebu-bio; Italy); more precisely, pEZ-M68 vector was employed for SLC3A2/4F2hc and pEZ-M29 for SLC7A7/y + LAT1, with the sequence encoding the eGFP positioned upstream of SLC7A7 ORF, so as to add the tag to the N-terminus of y + LAT1 protein.

The LPI_1_, LPI_2_, and LPI_4_ mutations of SLC7A7 were, then, obtained by means of Site Directed Mutagenesis on SLC7A7 ORF with the QuickChange II Site-Directed Mutagenesis kit (Agilent, Italy), according to the manufacturer’s instructions. The macro-deletion of LPI_3_ was, instead, reproduced through enzymatic digestion with BstBI upon a preliminary introduction of a second restriction site for the enzyme in position 499 of the ORF; the resulting sequences were separated on an electrophoretic gel and the band corresponding to the mutation-carrying plasmid was finally isolated.

The plasmid vectors obtained were, then, amplified through bacterial transformation in competent *E. coli* HB 101 and isolated using NucleoSpin® Plasmid kit (Macherey-Nagel GmbH&Co, Carlo Erba Reagents, Italy). After gene sequencing of the vectors obtained (Eurofins Genomics Srl, Itay), normal and mutant y + LAT1 were expressed in CHO cells through the simultaneous transfection with two plasmids (one carrying the w/t SLC3A2/4F2hc and, the other, either the w/t or the mutated version of SLC7A7/y + LAT1) by means of FuGENE® HD Transfection Reagent (Promega, Italy), according to the protocol provided; a transfection with empty vectors (one pEZ-M68 and one pEZ-M29) was performed in parallel to generate negative controls. For cell selection, CHO were maintained for 2 weeks in complete growth medium in the presence of 5 μg/ml Puromycin and 900 μg/ml Neomycin, then assayed for transport activity and cellular localization of y + LAT1.

### L-arginine influx

CAA transport in mammalian tissues is mediated by four distinct mechanisms, namely systems y^+^, y^+^L, b^0,+^, and B^0,+^ [6]. System y^+^ mediates a sodium-independent transport of cationic amino acids, while the other three systems accept both cationic (CAA) and neutral (NAA) amino acids [18]. System B^0,+^ is strictly Na^+^ dependent, whereas system b^0,+^ is Na^+^ independent; system y^+^L transports CAA in the absence of sodium and NAA in the presence of the cation.

For transport studies, monocytes and CHO cells were cultured onto 96-well trays. After two rapid washes in pre-warmed transport buffer (Earle’s Balanced Salt Solution, EBSS containing (in mM) 117 NaCl, 1.8 CaCl_2_, 5.3 KCl, 0.9 NaH_2_PO_4_, 0.8 MgSO_4_, 5.5 glucose, 26 Tris/HCl, adjusted to pH 7.4), cells were incubated for 1 min in the same solution containing [^3^H]arginine (50 μM, 5 μCi/ml) in the absence or presence of 2 mM leucine or 2 mM leucine + lysine. When sodium-independent transport was to be measured, a modified Na + −free EBSS (NMG-EBSS) was employed, with NaCl and NaH_2_PO_4_ replaced by N-methyl-D-glucamine and choline salts, respectively. The experiment was terminated by two rapid washes (< 10 s) in ice-cold 300 mM urea. The intracellular pool of cell monolayers was extracted in ethanol and radioactivity in cell extracts measured with Wallac Microbeta Trilux^2^ liquid scintillation spectrometer (Perkin Elmer, Wellesley, Ma, USA). L-arginine uptake was normalized for protein content, determined directly in each well by using a modified Lowry procedure [9]. Transport studies in lymphoblasts was, instead, determined as previously described [19]. Briefly, cells were centrifuged 5 min at 1100 rpm and the pellet was then suspended in EBSS. Aliquots of 100 μl, roughly corresponding to 2 × 10^6^ cells, were mixed to 100 μl of EBSS supplemented with ^3^H-Arg (100 μM, 4 μCi/ml). After 1 min the incubation was ended with a rapid dilution of the cell suspension in 2 ml ice cold 300 mM urea, followed by a centrifugation at 12,000 x g. The pellet was then washed with urea and extracted with 200 μl 5% sodium deoxycholate in 1 M NaOH. After 1 h, 100 μl of the cell extracts were used for radioactivity determination with a Wallac Microbeta Trilux^2^, while the remaining aliquot was used for protein determination with the modified Lowry procedure mentioned above.

Arginine uptake is expressed as nmol/mg of protein/min. System y^+^L activity was calculated as the difference between total uptake and the uptake obtained in the presence of 2 mM leucine in the presence of sodium; system y^+^ activity was calculated as the difference between the uptake measured in the presence of 2 mM leucine and in the presence of 2 mM leucine + lysine.

### RT-qPCR analysis

Total RNA was isolated with GeneJET RNA Purification Kit and reverse transcribed with RevertAid First Strand cDNA Synthesis Kit (Thermo Fisher Scientific, Italy); the expression of SLC7A6/y + LAT2, SLC7A7/y + LAT1 and that of the housekeeping gene RPL15 (Ribosomal Protein Like 15) were, then, monitored employing specific TaqMan® Gene Expression Assays (Thermo Fisher Scientific; Cat# Hs00187757_m1, Hs00909952_m1, and Hs03855120_g1, respectively), according to the manufacturer’s instructions. The amount of the gene of interest was expressed as numbers of mRNA molecules upon normalization to that of the housekeeping gene *RPL15* [20].

### Confocal microscopy

The subcellular localization of wild type (wt) and mutated y + LAT1 proteins was monitored in CHO transfected cells by means of confocal microscopy. To this end, cells were seeded on Glass Bottom Petri dishes (MatTek Corporation, USA) at a density roughly corresponding to 40% confluence; after 24 h, cells were washed twice with cold PBS, then incubated for 30 min at 4 °C with 5 μM BODIPY™ TR Ceramide (Thermo Fisher scientific, Italy) for the staining of Golgi apparatus in live cells. After three more washes with ice cold PBS, fresh growth medium was added to the plate and fluorescence was monitored with a confocal system (LSM 510 Meta scan, Carl Zeiss, Germany). eGFP (green) and BODIPY™ TR Ceramide (red) signals were acquired at 488 and 589 nm, respectively, and the emissions recorded through a 510 and 617 nm primary beamsplitter; regions of interest (ROI) of cells observed with a 40X (1.3 NA) oil-objective are shown.

### Statistics

The statistical analysis was performed by employing GraphPad Prism software; differences between groups have been evaluated by employing a Student’s t-test for paired samples and were considered significant when *p* < 0.05.

### Materials

Endotoxin-free fetal bovine serum (FBS) was purchased from EuroClone (Italy), while L-[2,3,4-^3^H]Arginine monohydrochloride (43 Ci/mmol) was obtained from Perkin-Elmer (Italy). Unless otherwise stated, Sigma-Aldrich (Italy) was the source of all the other chemicals.

## Results

### System y^+^L activity in LPI monocytes and lymphoblasts

**Fig. 1:**
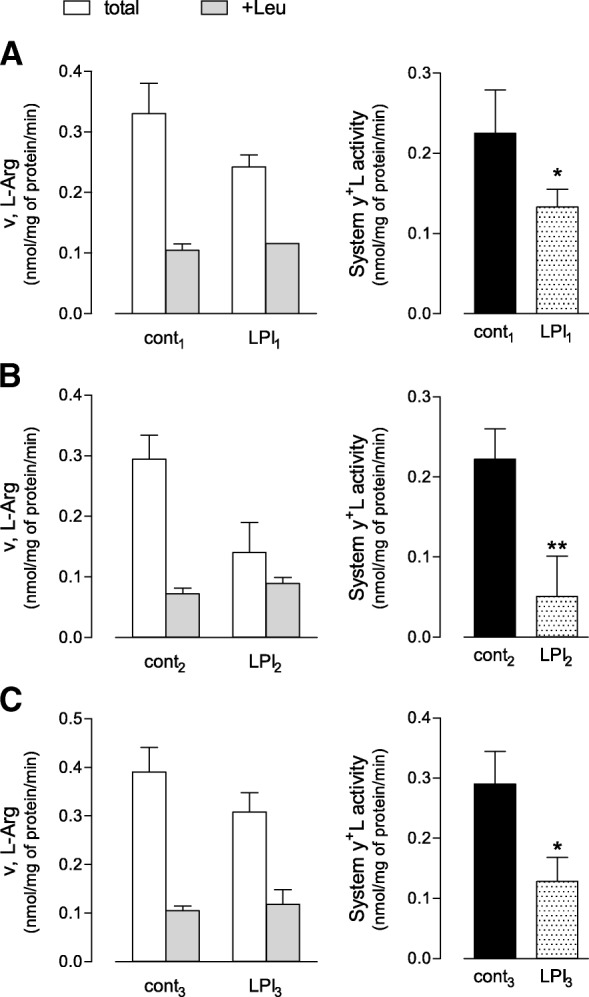
Transport activity of system y^+^L in monocytes from three LPI patients. Left Panels. Monocytes from healthy donors and LPI patients carrying different mutations of SLC7A7 were obtained as described in “Materials and Methods” (Panels **a**, **b** and **c** show results obtained for cont_1_ vs LPI_1_, cont_2_ vs LPI_2_, and cont_3_ vs LPI_3_, respectively). After a wash in EBSS, arginine uptake was assayed by 1-min incubation in the same solution supplemented with L-[^3^H]-arginine (50 μM; 4 μCi/ml) either in the absence or in the presence of 2 mM Leucine (see Methods). Data are means ± SD of four independent determinations in a representative experiment, repeated twice for each subject with comparable results. Right Panels. Data shown in left Panels were employed to calculate the relative contribution of system y^+^L as the difference between total transport and transport measured in the presence of leucine (see Methods). *p < 0.05, **p < 0.01 vs. control

Arginine transport through system y^+^L was measured in monocytes isolated from the three LPI patients enrolled and in three normal subjects age- and sex-matched. As expected, system y^+^L accounted for more than 80% of saturable arginine uptake in healthy monocytes (Fig. 1), confirming that this is the major transport system for arginine in these cells [21]. In cells isolated from all LPI patients, total arginine influx was lower than in normal monocytes (left Panels). The addition of leucine during the uptake assay reduced transport activity to values comparable in pathological and control monocytes; as a result, the *quota* of arginine transport referable to the activity of system y^+^L was significantly lower in any pathological cells than in normal controls (right Panels). The most evident reduction was observed in monocytes isolated from patient carrying c.1185_1188delTTCT mutation (LPI_2_, Panel b), although the impairment of arginine transport was significant also in LPI_1_ (Panel a) and LPI_3_ (Panel c).

**Fig. 2:**
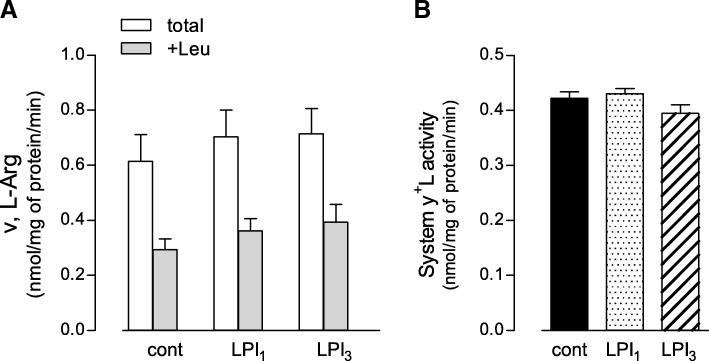
System y^+^L activity in lymphoblasts carrying the same mutations as LPI_1_ and LPI_3_. Panel **a**. Arginine transport was measured in lymphoblasts obtained from healthy donors (cont, *n* = 4) and LPI patients carrying LPI_1_ and LPI_3_ mutations (see “Materials and Methods”). Arginine uptake was assayed by 1-min incubation in EBSS supplemented with L-[^3^H]-arginine (50 μM; 2 μCi/ml), either in the absence or in the presence of 2 mM leucine (see Methods). For controls, data are means ± SEM of four different subjects; for LPI, data are means ± SEM of three independent experiments performed in cells obtained from the same subject. Panel **b**. Data shown in Panel **a** were employed to calculate the relative contribution of system y^+^L as the difference between total transport and transport measured in the presence of leucine (see Methods)

**Fig. 3:**
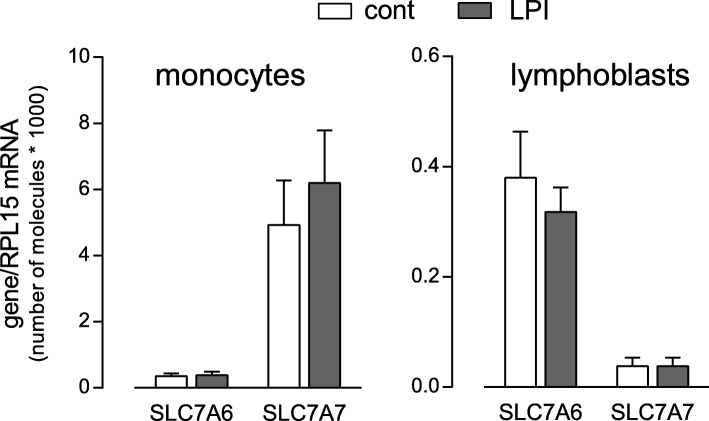
Expression of SLC7A6/y + LAT2 and SLC7A7/y + LAT1 in monocytes and lymphoblasts. The absolute amount of SLC7A6 and SLC7A7 mRNA (means ± SEM) were assessed in human monocytes (cont, *n* = 5; LPI, *n* = 3) and lymphoblasts (cont, n = 4; LPI, *n* = 2), as described in Methods

The effects of y + LAT1 mutations on arginine transport were also addressed in lymphoblasts obtained from other LPI patients sharing the same genetic defects as LPI_1_ and LPI_3_. Interestingly, contrary to what observed in monocytes, no significant difference was detected between normal and pathological cells in terms of neither total arginine transport (Fig. 2, Panel a) nor system y^+^L activity (Panel b). This result is consistent with the finding that, while SLC7A7 mRNA is expressed at significantly higher levels in monocytes than in lymphoblasts, the amount of SLC7A6 transcripts are comparable in the two cell models (Fig. 3); the amount of y + LAT1 and y + LAT2 transcripts is, thus, expected to give reason of the different effect of LPI defect on arginine transport in the two cell models.

### Characterization of arginine transport in CHO cells expressing LPI-causing mutations of SLC7A7

The LPI-causing mutations addressed in Figs. 1 and 2 were then reproduced in an in vitro model consisting of transfected CHO cells expressing y + LAT1 mutants, so as to assess their effects on both protein localization and function.

A preliminary characterization of arginine transport was performed in non-transfected cells, since no information is thus far available in this model. To this end, transport dependence on extracellular sodium was initially evaluated, so as to verify the contribution of the Na^+^/Cl^−^ dependent transport system B^0,+^ for neutral and cationic amino acids. In parallel, inhibition studies were conducted employing leucine as an inhibitor of systems y^+^L and b^0,+^ when added in the presence or in the absence of sodium, respectively. The complete inhibition of any saturable transport component was carried out through the further addition of an excess of lysine (2 mM) for the inhibition of system y^+^ activity, too.

**Fig. 4:**
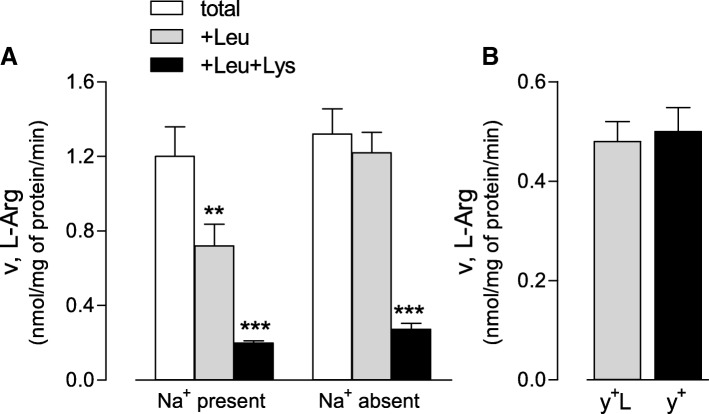
Characterization of arginine transport in CHO cells. Panel **a**. CHO cells, seeded in 96-well plates, were washed in EBSS in the presence or absence of sodium, and arginine transport was measured through a 1-min incubation in the same solution containing L-[^3^H] arginine (0.05 mM; 2 μCi/ml) in the absence or presence of 2 mM leucine (+Leu) or 2 mM leucine + 2 mM lysine (+Leu + Lys) (see Methods). Data are means ± SEM of three different experiments, each performed in quadruplicate. Panel **b**. Data shown in Panel **a** were employed to calculate the relative contribution of systems y^+^L and y^+^: the first corresponds to the difference between total transport and transport measured in the presence of leucine, while the latter to the difference between transport observed in the presence of leucine and that measured in the presence of leucine+lysine. ** p < 0.01, *** *p* < 0.001 vs control (total, Na^+^ present)

Results shown in Fig. 4, Panel a, indicate that total arginine transport was comparable in the presence and in the absence of sodium, thus excluding the contribution of system B^0,+^. The presence of leucine significantly decreased arginine transport when sodium was present, but not in its absence, demonstrating the presence of system y^+^L and ruling out the operation of system b^0,+^; the addition of lysine produced a further decrease of arginine transport, thus indicating a contribution of system y^+^ in these cells (Panel b).

**Fig. 5:**
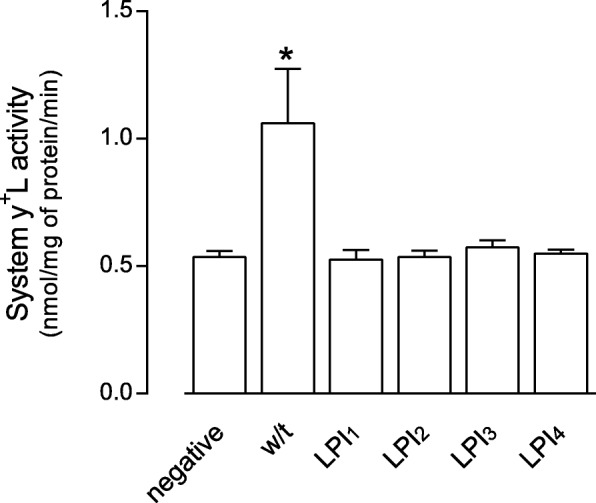
Effect of LPI mutations on arginine transport in transfected CHO cells. Transport activity of systems y^+^L was measured in CHO cells transfected with empty vectors (negative) and in cells transfected with plasmids carrying the indicated SLC7A7 mutations. y^+^L contribution was calculated as the difference between total transport and transport measured in the presence of 2 mM leucine. Data are means ± SEM of three different experiments, each performed in quadruplicate. * *p* < 0.05 vs negative

We next addressed the activity of y^+^L in CHO cells transfected with the plasmid bearing the wild-type (w/t) sequence of SLC7A7 or with the mutations either carried by the three LPI patients as in Fig. 1 (LPI_1_, LPI_2_ and LPI_3_) or described in literature, namely LPI_4_; CHO cells carrying empty vectors were employed for comparison (negative). As shown in Fig. 5, a significant increase of arginine transport through system y^+^L was observed in cells transfected with vectors carrying w/t SLC7A7, indicating the correct operation of the recombinant y + LAT1 protein upon CHO cell transfection. Conversely, when mutation-carrying plasmids were employed, arginine transport was comparable to that observed in negative-transfected cells, suggesting that all the SLC7A7 mutations studied hinder protein function.

### Analysis of the subcellular localization of w/t and mutated y + LAT1 proteins

**Fig. 6:**
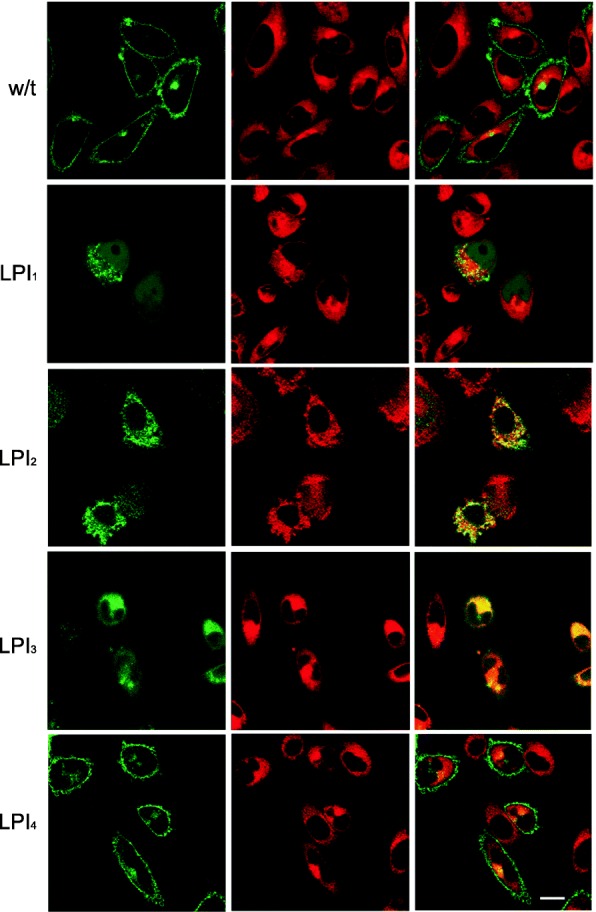
Confocal analysis of eGFP-tagged y + LAT1 mutants in CHO cells. CHO cells were transfected with plasmid vectors carrying the wild type (w/t) or mutated sequences of SLC7A7 (LPI_1–4_). *Left panels*: green signal due to eGFP-tag of y + LAT1 proteins; *central panels*: red signal obtained through cytosol staining with ceramide; *right panels*: overlapping of images in left and central panels, so as to address signals co-localization (yellow in merged images). Scale bar = 10 μm

Since no commercial anti-y + LAT1 antibody reliable for confocal microscopy is available, eGFP-tagged proteins were used to study the localization pattern of mutant transporters by means of confocal microscopy (Fig. 6 and in Additional file 1: Figure S1). Green and red signals in images correspond to eGFP-tagged y + LAT1 protein and cytoplasm staining by ceramide, respectively; when yellow signal was observed, this was due to the co-localization of y + LAT1 and Golgi apparatus.

As expected, cells transfected with the wild-type plasmid (w/t) showed a clear cut localization of the transporter on the plasma membrane. The same pattern of expression was observed in cells expressing c.1001 T > G mutation (LPI_4_), where y + LAT1 protein properly localized onto the plasma membrane; this is consistent with previous findings [14], and confirm the reliability of the protocol employed for the reproduction in vitro of patients’ defects.

Conversely, the signal given by the three eGFP-tagged mutants LPI_1_, LPI_2_, and LPI_3_ was evidently retained inside the cytosol, as confirmed by the appearance of a yellow staining, due to green and red signals co-localization. The pattern of expression of the transporter was, however, heterogeneous among the mutants; the green signal was, indeed, concentrated in vesicle-like structures in LPI_1_ (carrying the non-sense mutation c.726G > A), while it appeared patchy upon expression of the frameshift mutation c.1185_1188delTTCT (LPI_2_), and markedly widespread in LPI_3_ mutant cells, lacking exons 1–3 of SLC7A7 (c.del1–499).

## Discussion

In the present paper we address the effects of three LPI-causing mutations on y + LAT1 function and localization; to this extent, we took advantage of both ex vivo and in vitro cell models, i.e. circulating blood monocytes isolated from three LPI patients, lymphoblasts obtained from subjects carrying the same genetic defects, and CHO (Chinese Hamster Ovary) cells transfected with plasmid bearing the mutant proteins.

The mutations studied all determine important changes in SLC7A7 gene sequence, with LPI_1_ nonsense mutation (c.726G > A) leading to the loss of 6 out of 12 transmembrane protein domain, LPI_2_ (c.1185_1188delTTCT) causing a 15 bp forward shift of the stop codon, and LPI_3_ (c.1–499del) lacking 499 bp in 3′ region. The use of the in vitro model based on CHO transfected cells clarified that all these genetic defects translate into the lack of protein function (Fig. 5); although the precise localization and the fate of the mutant proteins in the intracellular compartment remains to be defined, the impairment of arginine transport is certainly ascribable to the cytosolic retention of the transporter (Fig. 6). Consistently, previous results obtained upon injection of mutant y + LAT1 proteins in *Xenopus* oocytes indicated that LPI frameshift mutations do not allow the transporter to reach the plasma membrane, either due to a premature degradation of the protein or to its retention into the reticular membrane [14]. Conversely, point mutation c.1001 T > G (LPI_4_) hinders arginine transport despite the correct localization of the protein onto the plasma membrane, suggesting either that this mutation affects the recognition site for the substrate or the translocation of the amino acid through the plasma membrane [14].

When employing ex vivo models, blood circulating monocytes isolated from the three LPI patients all displayed a significant, although incomplete, impairment of system y^+^L transport activity (Fig. 1); the residual transport activity observed is realistically ascribable to a basal activity of y + LAT2 transporter in these cells, since the localization studies performed in CHO transfected cells demonstrate that all the mutations addressed hamper the localization of y + LAT1 protein onto the plasma membrane (Fig. 6).

In contrast with results obtained in monocytes, no transport defect was observed in lymphoblasts obtained from other LPI subjects carrying the same mutations as LPI_1_ and LPI_3_ (Fig. 2), indicating that the transport defect differently manifests among cell types. To this concern, some years ago, we addressed the impact of an LPI-causing missense mutation (i.e., c.149 T > A) on arginine transport in different cell models obtained from the same Italian patient [9]. Results obtained demonstrated that system y^+^L activity was actually significantly compromised in circulating monocytes, but not in fibroblasts from the same subject; at molecular level, this difference was supposed to be due to the different expression of SLC7A7 gene in the two cells models, since it was much less abundant in fibroblasts than in monocytes. Similarly, in the present paper, we show that SLC7A7/y + LAT1 transcript is much more expressed in monocytes than in lymphoblasts despite a comparable expression of SLC7A6, coding for the light chain y + LAT2; no difference is observed between healthy and pathological cells (Fig. 3). These results, while confirming the primary role of SLC7A7/y + LAT1 in mediating arginine transport in monocytes, suggest that system y^+^L in lymphoblasts is mostly accounted for by y + LAT2 transporter, with an almost negligible contribution of y + LAT1; for this reason, the lack of a functional SLC7A7/y + LAT1 in LPI lymphoblasts has no effect on transport activity (Fig. 2). The present findings, moreover, corroborate our original hypothesis that the impact of LPI defect largely depends on the relative abundance of LPI target gene SLC7A7 in each cell model [9] and suggest that the typical affection of kidney, intestine, lungs and immune system in LPI may derive from an high expression and activity of SLC7A7/y + LAT1 in these districts.

As for the intestinal absorption and renal excretion of cationic amino acids, indeed, it is known that they are due to the activity of system y^+^L in the basolateral membrane of enterocytes and renal cells [7], where it is required for the efflux of CAA toward the bloodstream [22]. As a consequence, the decreased plasma levels and increased leakage in the urines of CAA, as observed in LPI patients, are usually referred to an impairment of transport activity consequent to the mutation of SLC7A7, implying that y + LAT1 is the transporter active in these cells; no study, however, has thus far addressed the relative expression of the genes coding for the two alternative light chains of system y^+^L in these tissues.

On the other hand, the high expression of SLC7A7 in innate immune cells ascribes a central role to these cells in the onset of the pulmonary and immunological complications of the disease, as suggested by many clinical and experimental evidences [23]. To this concern, we showed in a previous contribution that LPI defect associates with an impairment of the phagocytic activity of monocytes/macrophages from patients carrying the c.149 T > A missense mutation or the same mutations as LPI_1_ and LPI_2_ [8]. The original pathogenetic hypothesis for this finding was that the impairment of CAA efflux due to y + LAT1 defect in LPI monocytes/macrophages caused an increase of intracellular arginine concentration, leading to an increased production of the inflammatory mediator nitric oxide [2]. More recently, however, we proposed to include LPI in the group of auto-inflammatory diseases, given that SLC7A7 gene silencing in THP-1 monocytes induced the onset of an inflammatory phenotype, independently from the intracellular concentration of arginine [10]. Those results, by suggesting a thus far unknown immunomodulatory function of y + LAT1 protein, support the hypothesis of a central role for innate immune cells in the pathogenesis of LPI and its complications.

## Conclusion

The three LPI-causing mutations addressed for this study all hinder the correct localization of y + LAT1 protein onto the plasma membrane, hence causing a significant impairment of system y^+^L activity in circulating monocytes. However, despite the mutation, arginine uptake was comparable in lymphoblasts obtained from healthy subjects and LPI patients. The reason for this finding is proposed to be a differential expression of SLC7A7 among cell types, since high-expressing monocytes/macrophages typically manifest the transport defect, while low-expressing cells, such as fibroblasts and lymphoblasts, do not. In light of these results, we think that the relative abundance of y + LAT1 in LPI-target cells (such as renal and intestinal) deserves to be addressed, in the effort to ascertain the molecular mechanisms responsible for the clinical signs of LPI. In this context, the experimental approach adopted proved useful to deepen the cellular processing of the mutated y + LAT1 proteins so as to clarify some of the clinical features of LPI.

## Additional file

Additional file 1: **Figure S1.** Confocal analysis of eGFP-tagged y + LAT1 mutants in CHO cells. (PDF 484 kb)
