# Supplementary material for: Analysis of LPI-causing mutations on y+LAT1 function and localization
Source: Orphanet J Rare Dis. 2019 Mar 4;14:63. doi: 10.1186/s13023-019-1028-2 (PMC6399926; doi:10.1186/s13023-019-1028-2)
Supplement: Supplementary file 1 — Figure S1. Confocal analysis of eGFP-tagged y + LAT1 mutants in CHO cells. (PDF 484 kb) [file 13023_2019_1028_MOESM1_ESM.pdf]

Ms. OJRD-D-18-00494

Analysis of LPI-causing mutations on y+LAT1 function and localization

Bianca Maria Rotoli[1], Amelia Barilli[1], Filippo Ingoglia[1], Rossana Visigalli[1], Massimiliano G. Bianchi[1], Francesca Ferrari[1], Diego Martinelli[2], Carlo Dionisi-Vici[2], and Valeria Dall'Asta[1*].

Additional File 1

Figure 1S. Confocal analysis of eGFP-tagged y+LAT1 mutants in CHO cells. CHO cells were transfected with plasmid vectors carrying the wild type (w/t) or mutated sequences of SLC7A7 (LPI_{1-4}). *Left panels*: green signal due to eGFP-tag of y+LAT1 proteins; *central panels*: red signal obtained through cytosol staining with ceramide; *right panels*: overlapping of images in left and central panels, so as to address signals co-localization (yellow in merged images). Scale bar = 10µm

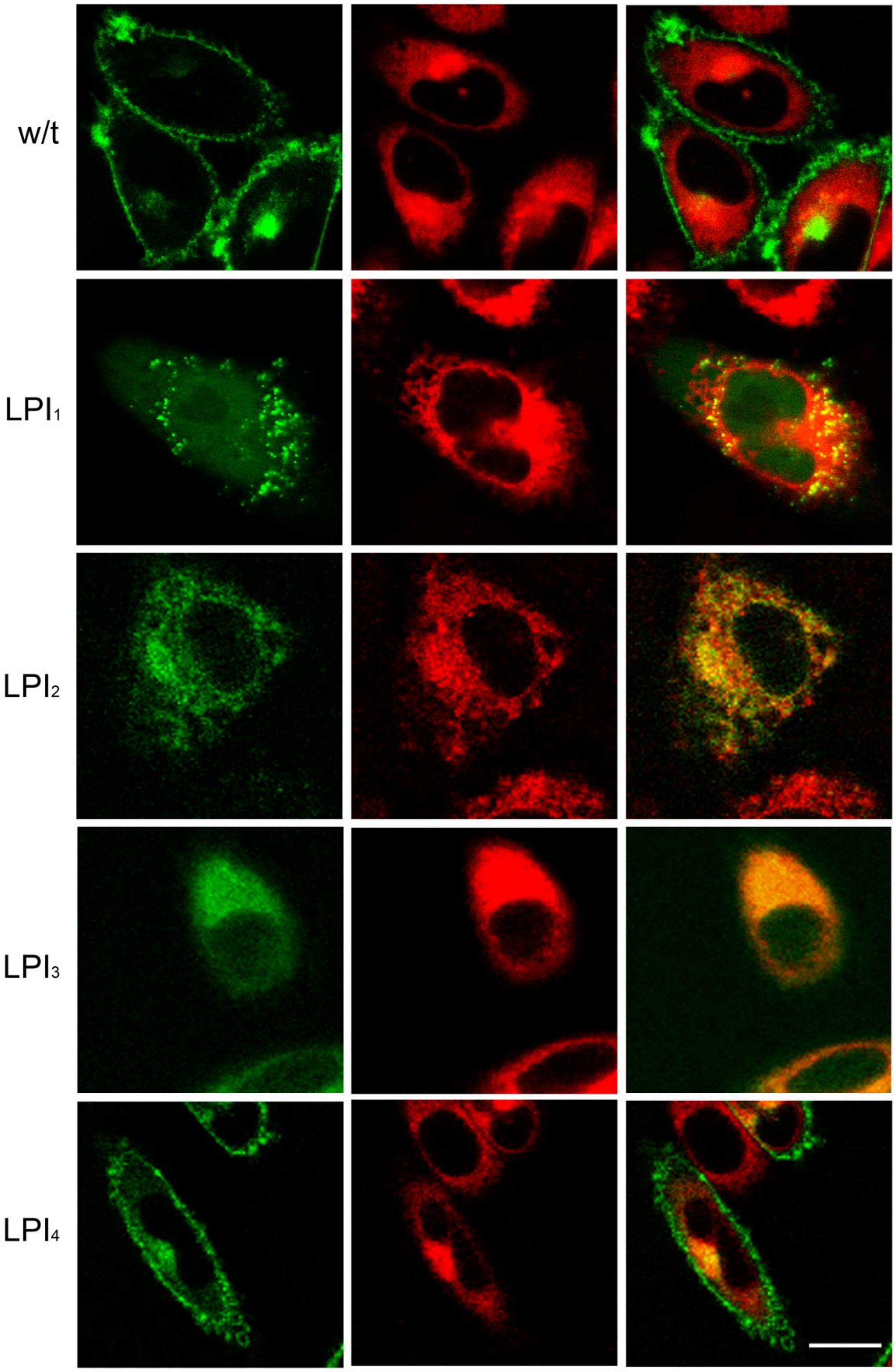

Figure 1S
